# Development of New Technologies for Risk Identification of Schistosomiasis Transmission in China

**DOI:** 10.3390/pathogens11020224

**Published:** 2022-02-08

**Authors:** Liang Shi, Jian-Feng Zhang, Wei Li, Kun Yang

**Affiliations:** 1 National Health Commission Key Laboratory of Parasitic Disease Control and Prevention, Wuxi 214064, China; shiliang@jipd.com (L.S.); zhangjianfeng@jipd.com (J.-F.Z.); liwei@jipd.com (W.L.); 2 Jiangsu Provincial Key Laboratory on Parasite and Vector Control Technology, Wuxi 214064, China; 3 Jiangsu Institute of Parasitic Diseases, Wuxi 214064, China; 4 Public Health Research Center, Jiangnan University, Wuxi 214064, China; 5 School of Public Health, Nanjing Medical University, Nanjing 211166, China

**Keywords:** schistosomiasis, risk identification, pathogen biology, immunology, 3S technology, mathematical modeling, molecular biology, big data, artificial intelligence, China

## Abstract

Schistosomiasis is serious parasitic disease with an estimated global prevalence of active infections of more than 190 million. Accurate methods for the assessment of schistosomiasis risk are crucial for schistosomiasis prevention and control in China. Traditional approaches to the identification of epidemiological risk factors include pathogen biology, immunology, imaging, and molecular biology techniques. Identification of schistosomiasis risk has been revolutionized by the advent of computer network communication technologies, including 3S, mathematical modeling, big data, and artificial intelligence (AI). In this review, we analyze the development of traditional and new technologies for risk identification of schistosomiasis transmission in China. New technologies allow for the integration of environmental and socio-economic factors for accurate prediction of the risk population and regions. The combination of traditional and new techniques provides a foundation for the development of more effective approaches to accelerate the process of schistosomiasis elimination.

## 1. Introduction

Schistosomiasis is one of the 20 neglected tropical diseases listed by the World Health Organization. It ranks second after malaria among the global human parasitic diseases in terms of socio-economic and public health importance in tropical and subtropical areas [1]. The estimated global prevalence of active infections is more than 190 million [2]. The main schistosome species in China is *Schistosoma japonicum*, and its snail host is *Oncomelania* *hupensis*. In the 1950s, China was among the countries with the heaviest schistosomiasis burdens, with more than 10 million patients, and schistosomiasis was endemic in 12 southern Chinese provinces. After nearly 70 years of arduous efforts, China’s schistosomiasis control program has achieved remarkable success. Especially in recent years, the number of schistosomiasis outbreaks has continued to decline in endemic areas, reaching the lowest level in history [3]. Up to 2020, based on the latest control and elimination criteria [4], of the 12 provinces (municipalities and autonomous regions) endemic for schistosomiasis in China, five provinces (Shanghai, Zhejiang, Fujian, Guangdong, and Guangxi) had achieved the criteria for elimination, two provinces (Sichuan and Jiangsu) had achieved the criteria for transmission interruption, and five provinces (Yunnan, Jiangxi, Hubei, Anhui, and Hunan) had achieved the criteria for transmission control. Among the 450 endemic counties (cities and districts) in the country, 337 (74.89%) met schistosomiasis elimination standards, 98 (21.78%) met transmission interruption standards, and 15 (3.33%) met transmission control standards [5].

Although China has made great advancements in the prevention and control of schistosomiasis, due to the wide distribution of endemic areas and complex environmental and socio-economic factors, schistosomiasis epidemic risk factors still exist: (i) international travel has resulted in imported schistosomiasis cases occasionally entering the country, a trend that is on the rise; (ii) many existing cases are found in patients with advanced schistosomiasis, while chronic infections are generally insidious manifestations of low-level infections [6]; (iii) control strategies for livestock sources of infection, such as sheep, dogs, and pigs, are weak [7], and wild animals have been found to be an occasional source of infection as well [8]; and (iv) *O. hupensis* is still widely distributed throughout the country and is affected by environmental and socio-economic factors such as global economic integration, climate change, frequent natural disasters, population movement, and wetland construction and ecological restoration [9,10,11,12,13,14,15,16,17]. Therefore, the control and elimination of schistosomiasis still faces many challenges.

Recently, risk assessment has become an important component of schistosomiasis control. Since 2016, the National Health Administration has organized multiple risk assessments using molecular biology techniques and other means to identify risk factors and at-risk areas, with the acknowledgement that epidemics are likely to rebound once schistosomiasis prevention and control strategies are relaxed [3]. As they advance from transmission control to transmission interruption and even elimination, schistosomiasis prevention and control strategies in China are changing their focus from extensiveness to precision. Therefore, risk assessment programs require more sensitive and accurate risk identification technologies [3,18]. Because complicated epidemiological, environmental, and socio-economic factors affect schistosomiasis transmission [19,20], many different technologies are used for schistosomiasis risk identification in different areas. Traditional risk identification technologies include pathogen biology, immunology, molecular biology, and imaging techniques. New risk identification technologies based on computer and communication technologies, including 3S technologies, mathematical modeling, big data, and artificial intelligence (AI), are also gradually being used in risk identification research. This study summarizes the application of traditional and novel technologies for risk identification and suggests priorities for technology development.

## 2. Applications of Traditional Risk Identification Technologies

Traditional risk identification technologies provide information for identifying epidemiological factors (patients, sick animals, *O. hupensis*, or infected *O. hupensis*) and provide a basis for predicting epidemics in large populations or assessing regional risk levels. Traditional risk identification technologies include pathogen biology, immunology, molecular biology, and imaging technology (Table 1).

### 2.1. Pathogen Biology Technologies

Pathogen biology technologies are used to detect schistosome eggs by microscopic examination of the stool or rectal tissues. Occasionally, they are used to detect schistosomiasis by observing hatching miracidia. A variety of convenient and cost-effective techniques have been developed, such as Kato–Katz (KK), thick smear, egg-hatch assays, and tissue biopsies [21,22,23].

Schistosomiasis is closely associated with the distribution of *O. hupensis*. Therefore, the identification of live *O. hupensis* or *O. hupensis* with cercariae is also an important risk factor. Live *O. hupensis* are observed through pathogen biology technologies. For example, *O. hupensis* can be observed with the naked eye to assess whether the soft body sticks out or crawls from the snail after standing still, or the shell of *O. hupensis* can be gently cracked by tapping to see whether the soft body moves within the shell [24]. Cercariae have often been observed in the liver tissue of crushed *O. hupensis* using a microscope. The sentinel method can also be used, in which mice are placed in water that may contain cercariae for a period of time. After 30–35 days, the mice are dissected to see if they are infected with schistosomiasis [25]. The nylon silk cloth or capron cloth fishing net method can be used to capture cercariae in water, and the principle of filtration has been used to design mechanical devices that enrich cercariae [26,27,28,29]. Sichuan Province carried out large-scale *O. hupensis* breeding site risk identification surveys every year from 2005 to 2015, with an average annual survey area of about 50,000 m^2^. Reservoirs and orchards were identified as major new *O. hupensis* habitats where there was a risk of schistosomiasis transmission [30]. During the flood disaster in 2020, Anhui Province and Wuhan City used traditional methods to identify risk factors such as the presence of *O. hupensis* in key areas, cercariae in bodies of water, and infections in free-range livestock and key personnel [31,32].

### 2.2. Immunological Technologies

Immunological technologies are based on the principles of immunity and identify whether humans or animals are infected with schistosomiasis by detecting anti-schistosome antibodies, schistosome antigens, or immune complexes. At present, the most commonly used immunological technologies in the field include indirect hemagglutination (IHA) tests, enzyme-linked immunosorbent assays (ELISAs), and colloidal dye test strips (DDIA) [31,33,34,35]. These methods are particularly useful when eggs cannot be identified in patients with light infections and can be used to quantify epidemics in different areas [36]. Jiangsu Province used DDIA to screen 2.382 million people at risk for schistosomiasis from 2006 to 2010, which played a huge role in implementing the “Schistosomiasis Control Strategy for Key Populations” and effectively controlling sources of schistosomiasis in Jiangsu Province [37].

### 2.3. Molecular Biology Technologies

Molecular biology technologies are increasingly being used in the early identification of schistosomiasis risk factors. These methods require only a small amount of nucleic acid, and a large number of target nucleic acid fragments can be obtained through amplification technologies, which can greatly improve detection sensitivity. Molecular biology techniques, such as polymerase chain reaction (PCR), have been used for risk identification and have a high sensitivity and specificity [38]. However, PCR requires residents to comply with strict testing protocols, and it involves complex laboratory testing methods. In recent years, constant temperature nucleic acid amplification technologies, such as loop-mediated isothermal amplification (LAMP), recombinase polymerase amplification (RPA), and recombinase-mediated isothermal amplification (RAA) have developed rapidly [39,40]. A highly sensitive and specific LAMP technology was established for the detection of serum-specific DNA in rabbits infected with schistosomiasis. The detection sensitivity was 100 times that of regular PCR [41]. This technology has now been widely used to detect schistosomiasis infections in live *O. hupensis* in the field [42,43,44]. The Jiangsu Institute of Parasitic Diseases has established two novel RAA technologies for the detection of schistosomiasis-specific gene fragments and *O. hupensis* infected with cercariae. Compared with traditional microscopy and PCR, RAA technology has the advantages of being fast, sensitive, and easy to operate [45,46].

### 2.4. Imaging Technology

The liver is the main parasitized and damaged target organ of schistosomes in humans. Schistosome eggs are deposited in the liver, where they cause granulomas, secondary liver fibers, and other changes. These lesions cause characteristic changes that can be detected by observation using imaging technologies. Imaging techniques, such as computed tomography (CT), ultrasonography (US), and magnetic resonance imaging (MRI), can also support the identification of schistosomiasis. For example, in previous studies, US was used to identify patients with schistosomiasis-associated liver disease in non-lake areas, CT was of great value in identifying patients with chronic hepatic schistosomiasis [47], and MRI was effective in identifying patients with schistosomiasis and assessing the severity of liver fibrosis [48].

In practice, traditional risk identification techniques are often used in combination and can be cross-referenced for improved accuracy. Researchers have used pathogen biology, IHA, and ELISA techniques to investigate the prevalence of schistosomiasis in local populations, migrant populations, and livestock in endemic areas in 10 counties (cities and districts) and in five provinces (cities), including Hubei, Jiangsu, Anhui, Shandong, and Chongqing. Schistosomiasis infections and the distribution of *O. hupensis* breeding locations were assessed using observational methods. The results showed that sources of infection and the risk of exogenous *O. hupensis* spread are increasing [49]. In Jiangxi, Hubei, and Anhui Provinces, rapid risk identification and evaluation of schistosomiasis transmission control standards using field observation, pathogen biology, and immunology technologies identified the activities of and infections in the population and cattle as the main risk factors [50,51,52,53].

## 3. Novel Risk Identification Technologies

**Figure 1:**
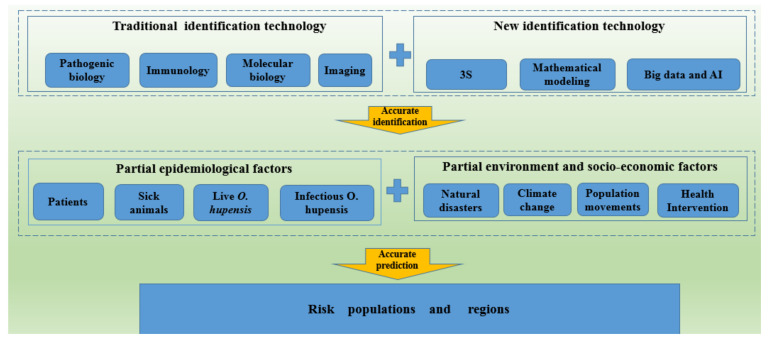
Schistosomiasis risk identification technologies.

With the development of computer technology, scholars are increasingly using 3S technology, mathematical modeling, big data, and AI to collect and analyze schistosomiasis epidemic data and environmental and socio-economic data to determine the relationship between risk factors and to identify at-risk areas or populations based on these factors (Table 1 and Figure 1).

### 3.1. 3S Technology

3S technology refers to integrated geographic information system (GIS), remote sensing (RS), and global positioning system (GPS) technologies. A large amount of accurate and real-time geographic environmental data (water bodies, surface type, artist index, surface temperature, soil, height and slope, etc.) can be obtained through RS, spatial information can be added to disease data using GPS, and visual representations of disease data and related environmental factors can be achieved using GIS. These tools allow for advanced analysis and data processing to describe disease distributions with greater accuracy, grasp the dynamics of disease development, identify risk areas, and develop disease control strategies. It is widely acknowledged that the frequency and transmission dynamics of schistosomiasis are closely related to environmental and socio-economic factors. The advantages of 3S technology have opened new avenues for risk identification research, such as identifying environmental risk factors and mapping risk prevalence areas, *O. hupensis* habitats, and transmission risks in relation to ecological transformations. For example, RS is used to identify environmental factors such as temperature, digital elevation model data, vegetation indices, distance from water, and other features of the study area. By combining these environmental factors with the epidemiological data of schistosomiasis patients, the distribution of snails can be further analyzed, and the distribution of schistosomiasis risk areas can be determined [54,55]. The environmental factor indicators obtained based on RS technology can be used to quantitatively explain the spatial variations in snail distribution and further establish risk and snail distribution prediction models to evaluate the risk of schistosomiasis transmission [56,57]. In recent years, with the rapid improvement of high resolution RS images, 3S technology has been applied to the identification of smaller spatial targets such as ditches in schistosomiasis endemic areas, the accurate analysis of spatial distribution relationships of risk factors for schistosomiasis infection, and rapid real-time identification of areas at risk for schistosomiasis transmission due to the spread of *O. hupensis* resulting from flooding [58,59].

### 3.2. Mathematical Modeling

Mathematical modeling for schistosomiasis risk identification mainly uses traditional identification and 3S identification technologies to identify various risk factors, determine the relationships between schistosomiasis and its influencing factors, and effectively integrate these factors to accurately identify high-risk areas or populations.

Hierarchical structure modeling is a common practical mathematical modeling technique to identify risk factors for the transmission of schistosomiasis. For example, a study in the Poyang Lake area using a hierarchical structure model found that schistosomiasis infections in humans and animals were the most important factor affecting the transmission of schistosomiasis [60]. In the South-to-North Water Diversion Project, a hierarchical model was used to find that snail breeding location had the greatest impact on the spread of schistosomiasis [61]. However, there are subjective differences when experts assign values to the importance of influencing factors, resulting in low reliability of research results. Regression models are also widely used to understand and identify risk factors, including single/multiple-level logistic regression models [62] and generalized linear models. Through regression analysis, weighted factors can be calculated to identify risk factors for schistosomiasis infection [63,64,65], which can help screen schistosomiasis risk identification factors and interpret the results. However, it is difficult to systematically and comprehensively understand the risk of schistosomiasis transmission. The transmission dynamics mathematical model studies the internal connections between components of the schistosomiasis transmission process, which assists in identifying the risk of schistosomiasis transmission. Based on schistosomiasis transmission studies in irrigated agricultural environments in western China, a transmission dynamics mathematical model was used to quantify environmental impacts on transmission intensity [66]. However, because transmission dynamics model assumptions are too ideal, such as the assumption that the spread of schistosomiasis is a closed system, the application of this model is restricted [67].

Spatial interaction and connectivity are important factors in the spread of schistosomiasis. Some classic models, such as regression models, only analyze the impact of a single factor or a few factors on the prevalence of schistosomiasis, require separate time or space dynamic analyses, and are seldom used to carry out a space analysis to ensure the accuracy of the model. Therefore, spatial and temporal dynamic analysis has become an important direction in schistosomiasis risk factor identification. Spatial and temporal analysis models analyze disease data from a spatial perspective by considering the relationship between spatial position and its related factors and the disease. Spatial autocorrelation and spatial scanning models are analysis methods for studying the spatial clustering of schistosomiasis and identifying at-risk areas. Spatial autocorrelation, both global and local, refers to correlations between attribute values of the same variable in different geographical locations and is used to measure whether attribute values of a given variable are spatially clustered [68]. For instance, global Moran’s I and Global Geary’s C were used to explore the spatial patterns of the distribution of snails on a small scale [69]. The spatial autocorrelation analysis revealed the existence of spatial clusters of human schistosomiasis infections and growing tendencies of spatial clustering over time. Spatial scanning technology is a method used to explore the location, size, and possibility of spatial aggregation in a research area [70]. Based on annual parasitological data recently collected at county and village levels, a multiscale spatiotemporal analysis was used to identify the transmission risk of *Schistosoma* *japonica* in Hunan Province from 2001 to 2015 in a GIS environment [71]. A spatial–temporal model of *S. japonica* transmission also employed a spatial interaction matrix based on neighborhood relationships and hydrologic connectivity to assess the effect of village parasite transport on schistosomiasis transmission and control [72]. SaTScan software was used to analyze time and space scanning statistics in Yunnan Province from 2004 to 2013 and revealed farm cattle and snail infection risk areas [73].

In order to better understand the temporal and spatial characteristics of schistosomiasis and to identify risk factors, spatial analysis models require more influencing factors to be analyzed. Commonly used spatial analysis models include time series models, spatial panel models, geographic weighted regression model (GWR), geographically and temporally weighted regression model (GTWR), Bayesian models, and niche models. The GWR model is a local spatial analysis method used mainly for non-stationary parameter estimation. It uses a specific bandwidth and distance-related weight function to fit a regression model at each geographic location [74].

The GTWR model is constructed by adding time effects to the GWR model to take into account spatiotemporal changes of the disease [75]. Combined with RS technology to obtain environmental factor data, GWR and GTWR models are used to identify the factors affecting the distribution of *O. hupensis* [76]. Bayesian models can be used to clarify temporal and spatial distribution patterns and changing trends in schistosomiasis transmission in an area through the analysis of temporal and spatial aggregation. At the same time, because the temporal and spatial effects of a particular region can be estimated by those of adjacent regions or time periods, this method can eliminate the influence of extreme values in some areas, making the curve of the risk distribution graph smoother and helping to identify high-risk areas or populations [77]. Bayesian models are increasingly being used to assess schistosomiasis risk, including identifying at-risk populations, determining *O. hupensis* distributions and high-risk areas, analyzing the impact of environmental factors [78,79,80], and developing schistosomiasis control strategies [81,82]. Niche models predict the distribution of a species by using its known distribution and related variables to analyze data, build a model, and extrapolate the results to different areas and time periods [54]. Hu et al. determined the risk of schistosomiasis transmission in Yunnan Province based on a niche model [83]. Fine-tuned Maxent models are also being used to anticipate distributions of *O. hupensis* in potential climate change scenarios. Model results indicate increased suitability for and range expansion of *O. hupensis* in the future [84].

### 3.3. Big Data and Artificial Intelligence Technology

The rapid advancement of computer and internet technologies is a driving force in the development and transformation of big data in schistosomiasis research. Researchers can analyze, evaluate, and address epidemic risks very quickly using big data collection, analysis, and mining techniques [85,86,87,88]. This can also facilitate accurate and effective health campaigns for the public in a timely manner and greatly improve early warning systems and responses to public health emergencies. Through the integration of environmental and socio-economic factor-related information systems and other public information resources, big data technologies can be used to comprehensively obtain data on relevant risk factors and, through deep mining and analysis, effectively evaluate the risk factors affecting schistosomiasis, allowing for the development of targeted prevention and control interventions [89]. Machine learning is the main solution to problems associated with big data analysis and mining. This can give computers the ability to discover potential patterns and features in data through algorithms, a method that has been used in risk predictions of schistosomiasis distribution weighted by spatial distance [90]. In addition, based on epidemic factors and related environmental factors, information combined with machine learning models (random forest, generalized boosted model) was used to identify and predict the distribution of schistosomiasis. Results showed that at-risk areas were mainly distributed in the coastal regions of the middle and lower reaches of the Yangtze River, the Poyang Lake region, and the Dongting Lake region [91].

AI technology is an important branch of information technology and has received increasing attention in medicine and public health [92,93,94]. As an important field of AI, computer vision and image recognition has been gradually applied to solve many problems caused by manual recognition in the prevention and control of schistosomiasis. Observing a large number of samples over a long period can cause eyesight fatigue and lead to misdetection. Image identification methods have been used to replace traditional methods of observing *Schistosome* miracidia, and they have the advantages of being highly sensitive and reproducible with a short detection time, high accuracy rate and low false positive and false negative rates [95]. The effectiveness of deep learning was confirmed in image identification tasks for the classification of *Bulinus* spp. and *Biomphalaria pfeifferi* snails and their parasite counterparts from the Senegal River in West Africa. That model achieved 99% and 91% accuracy for snail and parasite classifications, respectively [96]. An *O. hupensis* visual intelligence recognition model based on deep learning (convolutional neural network) was established to improve detection time and accuracy and reduce the amount of labor required for traditional *O. hupensis* identification techniques. The sensitivity, specificity, accuracy, Youden index, and F1 value of this model to identify *O. hupensis* were 91.00%, 97.50%, 96.20%, 88.50%, and 90.51%, respectively [97].

## 4. Lessons Learned in Risk Identification

Schistosomiasis risk identification research using both traditional and new identification technologies is growing. In order to achieve precise control of schistosomiasis, it is very important to select appropriate risk identification technologies.

Traditional identification technologies are the basis for the identification of schistosomiasis epidemiological risk factors and at-risk areas and populations. Among these technologies, pathogen biology methods are considered the “gold standard” for confirmation of schistosomiasis in China. However, these methods are time-consuming and laborious, and manual identification is subject to subjectivity, missed detection, and misjudgment, especially in areas where transmission has been interrupted or eliminated or where the infection rate and infectivity of people in endemic areas have been greatly reduced [98]. Immunological technologies are easy to operate and can be used for early detection of risk factors and quantitative identification of epidemics, all of which makes up for the shortcomings of pathogen testing to a certain extent. However, immunological technologies perform poorly in early schistosomiasis diagnosis and specificity and are not effective for the detection of low intensity infections, which challenges the accuracy and reliability of the identification of epidemic factors in endemic areas [99,100,101]. Molecular biology technologies have greatly improved the development of schistosomiasis risk factor identification methods, owing to their high specificity and sensitivity, and have laid a foundation for early risk screening in endemic areas with low schistosomiasis infection rates or low infectious snail densities [102]. However, molecular biology methods have high technical requirements and long detection times, which limits their application [103]. Imaging technology is widely used in hospitals to identify schistosomiasis and liver disease and is important for the identification of people at risk for advanced schistosomiasis. However, accuracy is easily affected by the technical skill of personnel, and there is often disagreement among observers [48].

The process of schistosomiasis transmission is complicated. Environmental and socio-economic factors, such as humidity, soil type, soil moisture, water flow, and health interventions, all influence the spread of schistosomiasis to varying degrees, especially as they impact the distribution of intermediate hosts [104,105,106,107]. Epidemiological factors alone may underestimate the risk of schistosomiasis transmission, especially in transmission interruption areas [3]. It is also difficult to quickly and accurately identify populations and regions at risk for schistosomiasis in real time and over large areas after natural disasters, climate change, and population movement, all of which affect the effective implementation of schistosomiasis control strategies [108]. Therefore, the study of novel technologies is of great importance to the accurate identification of factors affecting the prevalence of schistosomiasis and the precise identification of populations and areas at risk for schistosomiasis.

3S technology is the basis for spatial analysis and integrates a variety of technologies, including GIS, RS, and GPS, for the collection, sorting, and analysis of schistosomiasis data. With these technologies, data is rapidly updated, increasing the speed at which research can be done. The results are easily visualized, and schistosomiasis epidemic characteristics can be directly expressed. 3S technology provides a wealth of geographical and environmental data, which can be used for timely and appropriate identification of high-risk areas and to greatly improve identification accuracy [109], especially in areas that require prevention interventions but lack the means to monitor [110,111,112,113,114]. However, due to the wide variety of technical software used in 3S technology, collaboration between researchers and professionals with expertise in geography and RS is necessary. Mathematical models can be used to quantitatively screen multiple risk factors, reveal relationships between schistosomiasis and other factors, and predict which areas and populations are at risk. However, due to a lack of collaboration among various departments, it can be difficult for epidemiological researchers to obtain this type of risk factor data.

Traditional risk identification techniques are costly and require significant human and material resources. Factors such as reduced funding, rising labor costs, and aging personnel are also barriers to the application of risk identification technologies in areas where transmission has been interrupted [3]. The ideal technology should be more sensitive, significantly less expensive, and require less effort than any of the presently available technologies. Big data and AI technology provides new ideas for solving personnel-related problems in schistosomiasis prevention and control and is of great significance to the realization of accurate risk identification [115,116]. However, training of models requires massive amounts of data, environments for application scenarios are complex, and model stability and accuracy need to be further improved.

Table S1 summarizes studies that aimed to identify schistosomiasis risks using different technologies in the last 3 years in China. These studies reveal gaps in the following aspects: (i) less than half of the studies report risk identification research on environmental or socio-economic factors. Most studies identify only epidemiological risk factors for schistosomiasis, such as patients or *O. hupensis*. (ii) More than half of the studies identified risk using traditional techniques, which may have reduced sensitivity and accuracy. Traditional and new technologies each have their own advantages for risk identification, and the combination of these technologies for the identification of epidemiological, environmental, socio-economic, and other risk factors should be the focus of future research.

## 5. Conclusions

China has achieved schistosomiasis transmission control standards. The schistosomiasis infection rate in the most severely endemic areas has dropped from more than 10% at the beginning of this century to below 1%, and prevalence rates remain low [117]. However, risk factors affecting the spread of schistosomiasis still exist, and the risk of schistosomiasis resurgence remains a constant threat and a major obstacle to accomplishing transmission interruption in the country [118,119]. Thus, schistosomiasis risk identification is still a challenge. Therefore, molecular biology technologies should be rapidly developed with a focus on reducing costs, simplifying operations, shortening testing times, and further promoting the application of large-scale on-site testing. Large-scale schistosomiasis prevention and control programs involve multiple inputs and outputs as well as nonlinear and complex dynamic feedback systems, each of which is inter-related. It is necessary to accelerate the research and application of 3S, mathematical modeling, big data, and AI technologies and to combine traditional identification technologies to solve human-related issues and achieve accurate risk identification. In the future, with the development of computer network communication technology, these methods can be deployed on mobile devices at low cost and may greatly improve assessment and monitoring capabilities for schistosomiasis risk.

## Figures and Tables

**Table 1:** Technologies applied to schistosomiasis risk identification.

Technology	Applicable Risk Factors	Common Methods	Advantages	Limitations
Pathogen biology technologies	Epidemiological factors (patients, sick animals, live *O. hupensis* or cercariae)	Kato–Katz (KK), thick smear, egg hatch assay, tissue biopsy, etc.	Widely used in the field and considered the gold standard for the diagnosis of schistosomiasis	Time-consuming and laborious, and manual identification leads to errors due to subjectivity
Immunological technologies	Epidemiological factors (patients, sick animals, live *O. hupensis* or cercariae)	Hemagglutination test (IHA), enzyme-linked immunosorbent assay (ELISA), colloidal dye test strip method (DDIA), etc.	Low cost, convenient operation, convenient sampling, and quantitative identification of epidemics in different epidemic areas	Performs poorly in early diagnosis and specificity and ineffective for detection of low intensity infections
Imaging technologies	Epidemiological factors (schistosomiasis patients)	Computed tomography (CT), ultrasonography (US), magnetic resonance imaging (MRI), etc.	Auxiliary recognition of schistosomiasis is applied for the recognition of patients with schistosomiasis and liver disease	Accuracy is affected by the skill level of staff, and results of different observers often disagree
Molecular biology technologies	Epidemiological factors (patients, sick animals, live *O. hupensis* or cercariae)	Polymerase chain reaction (PCR), loop-mediated isothermal amplification (LAMP), recombinase polymerase amplification (RPA), recombinase-mediated isothermal amplification (RAA), etc.	Highly specific and sensitive, basis for early risk screening in endemic areas with low schistosomiasis infection rates or low infectious snail densities	Cost and technical requirements are high, detection time is long, and applications are limited
3S technologies	Environmental factors	Geographic information system (GIS), remote sensing (RS), and global positioning system (GPS)	Provides multiple methods for data collection, sorting, and analysis of schistosomiasis. Spatial data update speeds are fast, and study periods are short. Results are easily visualized, and schistosomiasis epidemic characteristics are directly expressed. Provides a wealth of geographical and environmental data for accurate mathematical modeling of populations and areas at risk for schistosomiasis.	Technical operations requires skilled professionals
Mathematical modeling	Epidemiological, environmental, and socio-economic factors	Hierarchical structure modeling, regression modeling, spatial autocorrelation modeling, spatial scanning modeling, geographic weighted regression modeling, geographically and temporally weighted regression modeling, Bayesian modeling, niche modeling, etc.	Used to study relationships between disease occurrence and other factors and to predict at-risk populations and areas	Difficulties in data collection for different risk factors
Big data and AI	Epidemiological, environmental, and socio-economic factors	Machine learning, image identification, deep learning, etc.	Accurately and quickly identifies risk factors and reduces labor costs, technical difficulties, and human judgment errors caused by subjectivity	Data demands are large, and identification reliability and accuracy need to be improved

## Data Availability

All data are available in the review.

## Supplementary Materials

The following supporting information can be downloaded at: https://www.mdpi.com/article/10.3390/pathogens11020224/s1, Table S1: Schistosomiasis risk identification-related papers within the last 3 years in China
