# Supplementary material for: Development of New Technologies for Risk Identification of Schistosomiasis Transmission in China
Source: Pathogens. 2022 Feb 8;11(2):224. doi: 10.3390/pathogens11020224 (PMC8877870; doi:10.3390/pathogens11020224)
Supplement: Supplementary file 1 [file pathogens-11-00224-s001.zip › pathogens-1514558-supplementary.pdf]

Table S1. Schistosomiasis risk identification-related papers within the last 3 years in China

NO.	Year	Reference	Study aim	Study areas	Scale	Technology	Range of risk factors
1	2021	[56]	To investigate *Oncomelania hupensis* breeding grounds and analyze breeding location and environmental preferences of snails; To identify schistosomiasis risk areas and explore the factors affecting occurrence and transmission of the disease	Dongting Lake Area	Meso	Traditional and new risk identification technologies	Environmental and socio-economic factors
2	2021	[108]	To create a model based on meteorological data to predict regions at risk of schistosomiasis during the flood season	China	Macro	Traditional and new risk identification technologies	Epidemiological and environmental factors
3	2021	[90]	To explore the feasibility of a random forest algorithm weighted by spatial distance for risk prediction of schistosomiasis distribution	Yangtze River Basin	Meso	Traditional and new risk identification technologies	Epidemiological and environmental factors
4	2021	[32]	To understand schistosome epidemics in high-risk areas experiencing flood damage	Anhui Province	Meso	Traditional risk identification technologies	Epidemiological factors
5	2021	[64]	To determine the incidence and prevalence of advanced schistosomiasis and risk factors associated with fatality in cases of advanced schistosomiasis	Hunan Province	Meso	Traditional risk identification technologies	Epidemiological factors
6	2021	[91]	To identify high-risk areas for schistosomiasis using information value and machine learning	China	Macro	Traditional and new risk identification technologies	Epidemiological and environmental factors
7	2021	[119]	To analyze risk monitoring data from 90 key schistosomiasis transmission risk monitoring villages and conduct a risk assessment	Wuhan City, Hubei Province	Micro	Traditional risk identification technologies	Epidemiological factors
8	2021	[31]	To assess the risk of schistosomiasis transmission after a flood	Wuhan City, Hubei Province	Micro	Traditional risk identification technologies	Epidemiological factors
9	2020	[14]	To evaluate the impact of flooding on schistosomiasis transmission along the Yangtze River basin	China	Macro	Traditional risk identification technologies	Epidemiological and environmental factors
10	2020	[59]	To use imaging data from radar remote sensing to extract water body information to perform rapid and effective identification of schistosomiasis transmission risk areas due to spreading of snails resulting from flooding	Dangtu County, Anhui Province	Micro	Traditional and new risk identification technologies	Epidemiological and environmental factors
11	2020	[44]	To assess schistosomiasis transmission risks	Deqiu Village, Deqiu Town and Baiyun Village, Nanjing Town, Yunnan Province	Micro	Traditional risk identification technologies	Epidemiological factors
12	2020	[25]	To assess the transmission risk of schistosomiasis after transmission interruption	Wuhan City, Hubei Province	Micro	Traditional risk identification technologies	Epidemiological factors
13	2020	[83]	To predict the transmission risk of schistosomiasis based on ecological niche modeling and to identify high-risk areas	Yunnan Province	Meso	Traditional and new risk identification technologies	Environmental and socio-economic factors

14	2020	[38]	To develop specific interventions for the population, assess knowledge, attitudes and practices (KAPs) of fishermen and boatmen towards schistosomiasis, and to identify the risk factors associated with schistosome infection using molecular techniques	Yueyang County, Hunan Province	Micro	Traditional risk identification technologies	Epidemiological factors
15	2019	[27]	To evaluate potential transmission risks of schistosomiasis as evidence to plan measures for schistosomiasis prevention and control	Yunnan Province	Meso	Traditional risk identification technologies	Epidemiological factors
16	2019	[43]	To evaluate the potential risk of schistosomiasis transmission as evidence for formulating control strategies	Chuxiong City, Yunnan Province	Micro	Traditional risk identification technologies	Epidemiological factors
17	2019	[54]	To identify and monitor potential schistosomiasis risk areas in the Dongting Lake area using remote sensing (RS) and geographic information system (GIS) technology	Dongting Lake area	Meso	Traditional and new risk identification technologies	Epidemiological and environmental factors
18	2019	[17]	To clarify the dominant climate and ecological factors affecting the risk of schistosomiasis transmission based on a maximum entropy model	China	Macro	Traditional and new risk identification technologies	Environmental factors
19	2019	[65]	To derive and validate a risk prediction model to be applied in clinical practice	Jingzhou and Huangshi, two prefecture-level cities in Hubei Province	Micro	Traditional and new risk identification technologies	Epidemiological factors
20	2019	[109]	To use high-resolution remote sensing imaging to extract ecological factors and to model and predict potential schistosomiasis risk areas	Dongting Lake area	Meso	Traditional and new risk identification technologies	Epidemiological and environmental factors
21	2019	[73]	To explore the spatial-temporal clustering of schistosomiasis transmission risk	Yunnan Province	Meso	Traditional and new risk identification technologies	Epidemiological factors
22	2019	[16]	To build a schistosomiasis transmission risk surveillance system	Sichuan Province	Meso	Traditional risk identification technologies	Epidemiological factors
23	2019	[23]	To analyze risk factors for schistosomiasis transmission	Mianyang City, Sichuan Province	Micro	Traditional risk identification technologies	Epidemiological factors
24	2019	[42]	To discover the risk of schistosomiasis transmission	Ma'anshan City, Anhui Province	Micro	Traditional risk identification technologies	Epidemiological factors
25	2019	[24]	To carry out risk monitoring of schistosomiasis transmission in key villages and provide a scientific basis for formulating control countermeasures	Wuhan City, Hubei Province	Micro	Traditional risk identification technologies	Epidemiological factors
